# Supplementary material for: Knowledge, beliefs, attitudes and perceived risk about COVID-19 vaccine and determinants of COVID-19 vaccine acceptance in Bangladesh
Source: PLoS One. 2021 Sep 9;16(9):e0257096. doi: 10.1371/journal.pone.0257096 (PMC8428569; doi:10.1371/journal.pone.0257096)
Supplement: S1 Questionnaire — (DOCX) [file pone.0257096.s001.docx]

**COVID-19 Vaccine Acceptability Survey**

# Consent

Assalamu Alaikum/Adab (Greetings). I would like to request you to take part in a small survey. The purpose of this study is to understand your attitude toward the COVID-19 vaccine(s) in Bangladesh. The survey includes questions regarding your knowledge and attitude towards COVID-19 and COVID-19 vaccines in Bangladesh. It also includes some socio-demographic questions. I hope this information will help the Bangladesh government and policymakers to make a better vaccination strategy to fight off the COVID-19 pandemic. You will not be asked any personal or sensitive questions. You will not be asked your name/identity. The survey may take 4-5 minutes to complete. Please note that your participation in the survey is completely voluntary. You can reject it anytime during the survey.

Are you willing to participate in this survey?

1. Yes
2. No

If the answer is ”NO”, then shows “Thank you for your time” and ends the survey. Otherwise, the survey continues.

| **Section A** | **Demographic** | | |
| --- | --- | --- | --- |
| **Serial No.** | **Question** | **Options** | **Remarks** |
| 1 | (**Eligibility Check**)  How old are you (in years)? | 1. 17 or younger 2. In between 18 to 29 3. In between 30 to 50 4. In between 51 to 70 5. In between 71 to 100 | If the answer is ”1” then shows “Unfortunately you are not eligible to take part in this survey as you are under 18, please stop and submit it” |
| 2 | (**Eligibility Check**)  Do you currently live in Bangladesh? | 1. Yes 2. No | If the answer is ”No” then shows “Unfortunately you are not eligible to take part in this survey as you are not Bangladeshi, please stop and submit it” |
| 3 | How would you describe your gender? | 1. Female 2. Male 3. Other(specify) |  |
| 4 | Where do you live? | 1. Urban (City corp, municipality) 2. Rural |  |
| 5 | What is your level of education? | 1. HSC/Alim/ Vocational/Nursing or less 2. University degree (Hon's/MBBS/ Masters 3. Honors or MBBS |  |
| 6 | What is your religion? | 1. Islam 2. Hindu 3. Buddhist 4. Christianity 5. Other religion, please describe |  |
| 7 | In which of the following categories would you place your monthly household income from all sources before tax and any other deductions? | 1. Under 30,000 2. 30,000–39,999 3. 40,000–49,999 4. 50,000–74,999 5. 75,000 or over 6. Don't know |  |
| 8 | What is your **marital** status? | 1. Unmarried 2. Married or civil union 3. Divorced 4. Separated 5. Widowed |  |
| 9 | What is your occupational Status? | 1. service holder (govt/private) 2. Entrepreneur/business 3. Student 4. Housewife/Retired/Unemployed/ Other) |  |
| 10 | Did you take any vaccine after 18 years of age? | 1. Yes 2. No |  |

| **Section B** | **Knowledge and Belief** | | |
| --- | --- | --- | --- |
| **Serial No.** | **Question** | **Options** |  |
| **1** | Is COVID-19 a lethal infectious disease? | 1. Yes 2. No 3. Don’t know |  |
| **2** | Is COVID-19 deadlier for elderly people (60+ years)? | 1. Yes 2. No 3. Don’t know |  |
| **3** | Do mostly elderly and sick people die of COVID-19? | 1. Yes 2. No 3. Don’t know |  |
| **4** | Can COVID-19 not spread from one to another by contact? | 1. Yes 2. No 3. Don’t know |  |
| **5** | Are hot and humid countries like Bangladesh safe from COVID-19? | 1. Yes 2. No 3. Don’t know |  |
| **6** | Is it human-made and deliberately released? | 1. Yes 2. No 3. Don’t know |  |
| **7** | Was the COVID-19 virus genetically engineered as part of a biological weapons program? | 1. Yes 2. No 3. Don’t know |  |
| **8** | Is this a normal disease like cold/cough and fever? | 1. Yes 2. No 3. Don’t know |  |
| **9** | Do people recover from it without any treatment? | 1. Yes 2. No 3. Don’t know |  |
| **10** | Is COVID-19 caused by the same virus that causes influenza (flu)? | 1. Yes 2. No 3. Don’t know |  |
| **11** | Can Testing help people determine if they are infected with SARS-CoV-2, what do you think? | 1. Yes 2. No 3. Don’t know |  |
| **12** | Is there any effective medicine available for treating COVID-19/ coronavirus? | 1. Yes 2. No 3. Don’t know |  |
|  | **Attitude and Belief**  How much do you agree with the following statements | | |
| 13 | Vaccination is an effective way to prevent and control a disease | 1. Agree 2. Neutral 3. Disagree |  |
| 14 | Young (less than 30) and children do not need any vaccination against COVID 19 | 1. Agree 2. Neutral 3. Disagree |  |
| 15 | We need to prioritize going back to our normal routines (opening schools, colleges, offices) as soon as possible by maintaining safety protocols. | 1. Agree 2. Neutral 3. Disagree |  |
| 16 | It should be a crime if people know that they have COVID-19 but they don’t isolate them | 1. Agree 2. Neutral 3. Disagree |  |
| 27 | The Covid-19 vaccines that are being inoculated worldwide are effective and safe | 1. Agree 2. Neutral 3. Disagree |  |
| 18 | Vaccines should be marketed and distributed entirely by the government in Bangladesh- what do you think? | 1. Agree 2. Neutral 3. Disagree |  |
|  | **Perceived Barrier and Perceived Risk** | | |
| 19 | **(Perceived Barrier)**  If I decided to get the COVID-19 vaccine, it would be hard to find a provider or clinic that could give me the vaccine. | 1. Agree 2. Neutral 3. Disagree |  |
| 20 | **(Perceived Barrier)**  The COVID-19 vaccine might have side effects. | 1. Agree 2. Neutral 3. Disagree |  |
| 21 | **(Perceived likelihood)**  What do you think is the chance that you will get COVID-19 in the future? | 1. No chance 2. Low Chance 3. Medium chance 4. High chance |  |
| 22 | **(Perceived severity)**  How severe do you think it would be if you get COVID-19? | 1. Not at all 2. Low Chance 3. Medium 4. Very severe |  |

| **Section C** | **Vaccine acceptability** | | |
| --- | --- | --- | --- |
| **Serial No.** | **Question** | **Options** |  |
| 1 | Have you heard of any vaccine that is going to be inoculated in Bangladesh? | 1. Yes 2. No |  |
| 2 | Bangladesh Govt. is going to inoculate the COVID-19 vaccine, will you take it? | 1. Yes 2. No |  |
| 3 | Why are you doubtful about taking the vaccine?  (Apply all that apply) | 1. Not sure about its efficacy 2. Doubtful about its side effects or safety 3. COVID-19 is going away 4. I am young and don’t need it 5. The vaccine is coming from India 6. I don’t trust vaccination 7. Don’t know | If Q2 is “NO”, answer this question |
| 4 | When will you or your family members take the vaccine? | 1. After 2-6 months if seems safe and effective 2. If COVID-19 becomes deadlier in Bangladesh 3. Don’t know | If Q2 is “Yes”, answer this question |
| 5 | What should be the price of a complete dose of a vaccine? | 1. Should be free 2. 1-1000 3. 1000+ |  |
| 6 | Considering the current scenarios, who do you think should receive the first shipment of the vaccine in Bangladesh? | 1. Healthcare workers/professionals 2. Elderly people (60+ years) 3. People who have underlying diseases 4. Politicians 5. Other (specify) |  |
| **Closing Statement** | **That completes the survey. Thank you for your valuable time!** | | |
