# Supplementary material for: Knowledge, beliefs, attitudes and perceived risk about COVID-19 vaccine and determinants of COVID-19 vaccine acceptance in Bangladesh
Source: PLoS One. 2021 Sep 9;16(9):e0257096. doi: 10.1371/journal.pone.0257096 (PMC8428569; doi:10.1371/journal.pone.0257096)
Supplement: Attachment — Submitted filename: Response to Reviewers.docx [file pone.0257096.s002.docx]

August 21, 2021

Editor in Chief

Emily Chenette

PLOS ONE

Subject: Submitting Revised Manuscript [PONE-D-21-11048] for the journal “PLOS ONE”

Title: Knowledge, beliefs, attitudes and perceived risk about COVID-19 vaccine and determinants of COVID-19 vaccine acceptance in Bangladesh

Dear Editor,

Thank you for allowing me to resubmit the revised manuscript. We appreciate the time and effort that you and the reviewers have dedicated to providing us with your valuable feedback on our manuscript. We are truly grateful to the reviewers for their insightful comments on our paper. We believe these suggestions will help improve the quality of the manuscript. We have been able to incorporate changes to reflect all the suggestions provided by the reviewers. We have highlighted the changes within the manuscript by using Track Changes. We have also provided an unmarked version of our revised paper without tracked changes.

Here is the point by point responses to the reviewers’ comments and concerns:

**Response to Editor:**

**Editor point #1:** “Please ensure that your manuscript meets PLOS ONE's style requirements, including those for file naming. The PLOS ONE style templates can be found at

https://journals.plos.org/plosone/s/file?id=wjVg/PLOSOne_formatting_sample_main_body.pdf and

https://journals.plos.org/plosone/s/file?id=ba62/PLOSOne_formatting_sample_title_authors_affiliations.pd”

**Author response #1:** We would like to thank you for your suggestion. We have separated the main body and the title pages of the manuscript and these two files have been organized based on the instructions.

**Editor point #2:** “Please review your reference list to ensure that it is complete and correct. If you have cited papers that have been retracted, please include the rationale for doing so in the manuscript text, or remove these references and replace them with relevant current references. Any changes to the reference list should be mentioned in the rebuttal letter that accompanies your revised manuscript. If you need to cite a retracted article, indicate the article’s retracted status in the References list and also include a citation and full reference for the retraction notice.”

**Author response #2:** Thank you for the legit suggestion. We have rechecked the reference list it is complete and correct.

**Editor point #3:** “Please revise your tables to replace p-values of "0.000" to "<0.001”

**Author response #3:** Thank you for the suggestion. It has been done.

**Editor point #4:** “Thank you for stating the following financial disclosure:

"The funders had no role in study design, data collection and analysis, decision to publish, or preparation of the manuscript."

At this time, please address the following queries:

a) Please clarify the sources of funding (financial or material support) for your study. List the grants or organizations that supported your study, including funding received from your institution.

b) State what role the funders took in the study. If the funders had no role in your study, please state: “The funders had no role in study design, data collection and analysis, decision to publish, or preparation of the manuscript.”

c) If any authors received a salary from any of your funders, please state which authors and which funders.

d) If you did not receive any funding for this study, please state: “The authors received no specific funding for this work.”

Please include your amended statements within your cover letter; we will change the online submission form on your behalf.”

**Author response #4:** Since we did not receive any funding for this study, we have stated “The authors received no specific funding for this work.”

**Response to Reviewer #1:**

**Reviewer point #1:** “Dear author,

Please add population size, age range & ethical approval number from organization in the abstract

Concise the introduction its too lengthy

There is no need to mention results again in discussion only discuss your results with previous studies

Make the article more concise and compact”

**Author response #1:** We would like to thank you for these detailed comments. We have added population size, age range, and details regarding ethical approval in the abstract. We have refurbished the introduction and discussion to make them more succinct. We have removed the redundant parts from the discussion section.

**Response to Reviewer #2:**

**Reviewer point #1:** “The study performed is current with sound idea and findings. The methodology and results are well presented in manuscript. The work is well appreciated for collection and presentation of data. The title is very simple, its must be elaborated.”

**Author response #1:** Thank you for your appreciation and encouraging words. As you suggested we have elaborated the title of this manuscript.

In addition, we went through the whole article for a similar types of typos and editing mistakes. We believe that the changes have improved the revised manuscript, which you will find updated.
